# Comparative Analysis of Woody Biomass Fly Ash and Class F Fly Ash as Supplementary Cementitious Materials in Mortar

**DOI:** 10.3390/ma17153723

**Published:** 2024-07-27

**Authors:** Yaru Yang, Koji Takasu, Hiroki Suyama, Xiangnan Ji, Murong Xu, Zihao Liu

**Affiliations:** 1 Architecture Course, Graduate School of Environmental Engineering, The University of Kitakyushu, 1-1 Hibikino Wakamatsu, Kitakyushu 808-0135, Fukuoka, Japan; e3dbb418@eng.kitakyu-u.ac.jp (Y.Y.); m15822762154@163.com (X.J.); d2dbb409@eng.kitakyu-u.ac.jp (M.X.); 2 Department of Architecture, Faculty of Environmental Engineering, The University of Kitakyushu, 1-1 Hibikino Wakamatsu, Kitakyushu 808-0135, Fukuoka, Japan; suyama@kitakyu-u.ac.jp (H.S.); z-liu@kitakyu-u.ac.jp (Z.L.)

**Keywords:** biomass fly ash, pozzolanic reaction, compressive strength, drying shrinkage, micro-pore structure

## Abstract

Biomass fly ash is a sustainable, eco-friendly cement substitute with economic and performance benefits, being renewable compared to coal fly ash. This study examines using biomass fly ash (BFA) as a sustainable cement substitute, comparing it with Class F fly ash (CFA). With a water–binder ratio of 0.5 and replacement rates of 10%, 15%, 20%, 25%, and 30% (by mass), the research highlights BFA’s promising applications. BFA and CFA were mixed into cement paste/mortar to analyze their reactivity and properties, with hydration products CH and C-S-H evaluated at 7, 28, and 91 days. Compressive strength, micro-pore structure, and drying shrinkage (assessed from 7 to 182 days) were tested. Results showed BFA had similar pozzolanic reactions to CFA at later stages. While compressive strength decreased with higher BFA replacement rates, early-stage performance matched CFA; growth was CFA-10 (18 MPa) and BFA-10 (17.6 MPa). BFA mortars exhibited slightly better deformation properties. BFA-30 cement had superior performance, with a lower drying shrinkage rate of 65.7% from 14 to 56 days compared to CFA-10’s 73.4% and a more stable shrinkage growth rate decrease to 8.4% versus CFA-10’s 6.4% after 56 days. This study concluded that BFA, usable without preprocessing, performed best at a 10–15% replacement rate.

## 1. Introduction

The construction industry is thriving with societal progress but faces significant environmental challenges from raw material extraction, processing, transportation, and use. Current policies and business trends show ecological considerations are driving the sector to reduce reliance on unsustainable resources and minimize CO_2_ emissions throughout the life cycle [1]. The high demand for construction materials has made alternative materials a favorable option, mitigating environmental impacts and offering economic benefits. Since its introduction in the 1820s, Ordinary Portland Cement (OPC) has provided essential strength to concrete structures. However, its increasing use has raised environmental concerns [2,3,4]. Extensive literature suggests that there is substantial potential within the cement industry to reduce greenhouse gas emissions and alleviate environmental stress [5,6,7]. Researchers are increasingly interested in supplementary cementitious materials (SCMs) produced from various raw materials for use as concrete substitutes [8,9,10,11,12,13]. Fly ash, a by-product of coal power plants, is widely researched for its ability to replace cement [14,15], reducing cement consumption and environmental impact, which results in lower costs and carbon emissions [16]. Some biomass-burning fly ash also shows pozzolanic activity [17,18], making it a cleaner, more environmentally friendly SCMs.

Biomass fly ash, from agricultural wastes, paper production, and other bioenergy by-products, reduces biomass waste and fossil fuel consumption [19]. Its low carbon content, low CO_2_ emissions, and low sulfur chain are non-negligible advantages. As environmental awareness grows, coal power plants are being phased out, increasing demand for clean, renewable energy and greater use of biomass energy [20]. Since the introduction of Japan’s Renewable Energy Feed-in Tariff (REFIT) program in July 2012, numerous wood biomass power plants have been planned nationwide, with 58 operational plants expected by 2017. It is projected that these wood-fired boilers will generate at least 50 million tons of combustion ash annually (referred to as wood-fired ash). Furthermore, with over 100 operational power plants anticipated across Japan by 2025, it is estimated that an additional 80 million tons of wood combustion ash will be generated [21]. Considering the declining reserves of high-quality SCMs, it becomes crucial to produce alternative SCMs such as BFAs on a large scale for their application in the concrete industry.

The use of biomass energy produces significant biomass fly ash. Exploring eco-friendly ways to manage and utilize these by-products offers promising opportunities. Several researchers have already investigated the application of biomass fly ash in the cement industry [22,23,24,25,26]. Wang S, Baxter L, and Fonseca F analyzed concrete samples with 25% cement replaced by fly ash from woody combustion [27]. They used SEM, EDX, and ESEM to assess the microstructure and pozzolanic reaction progress. After one year, the concrete showed a product ring around many particles with inert cores. The sample displayed a laminar flow structure identified as calcium silicate hydrate gel via EDX, resulting from hydration reactions. Finer particles in fly ash demonstrated higher pozzolanic reactivity due to their larger specific surface area [27]. Teixeira et al. [28] investigated the carbonization and hydration effects resulting from using woody combustion fly ash in construction materials. When fly ash is incorporated, the overall system’s reactions become more complex than when OPC is the sole ingredient. Fly ash engages in a pozzolanic reaction with the Ca(OH)_2_ produced by the hydration of the cement clinker, in addition to the hydration of the clinker itself upon contact with water [29]. However, biomass fly ash has a different mechanism due to variations in chemical composition among batches and differences in particle size distribution and specific surface area at a microscopic level [30]. Previous studies by Demir İ, Sevim Ö, Filazi A. et al. confirmed that the particle size distribution of pozzolanic material (FA) affects the hydration reaction [31]. Fly ash admixed cement mortar with optimized particle size distribution also had a high degree of compactness after 28 and 90 days. The pozzolanic effect of fly-ash-blended cement mortar was also enhanced with increasing dosage, thus improving the durability [32]. In another study in the literature [33], it was shown that the strength measurements of cementitious composite mortar with optimized particle size distribution of fly ash were higher than those of unoptimized fly ash at the same substitution rate. It can be concluded that optimizing the particle size distribution of fly ash ensures the filler effect of cementitious composite systems. During the process of hydration reaction, heterogeneous nucleation and growth of calcium silicate hydrate (C-S-H) are facilitated not only by fly ash providing additional surfaces (filler effect) but also through a gelling reaction with one of the hydration products (portlandite), resulting in additional C-S-H formation [34]. In addition, during hydration, the sulfate/alumina ionic ratio of the biomass fly ash generally favors the formation of ettringite at an early stage and monosulfoaluminate at a later stage, which has implications for the development of strength [35]. In contrast to ordinary coal fly ash, incorporation of biomass fly ash leads to lower levels of concrete hydration possibly due to its lower content of chemically bound water. Regarding calcium hydroxide consumption, biomass fly ash exhibits comparable capabilities as coal fly ash. The focus of our research lies on the hydration reaction since it provides essential strength for concrete building materials within this comprehensive framework concerning fly ash investigation. Apart from cement clinker’s hydration process upon contact with water, we can refer to the pozzolanic reaction involving the participation of pozzolanic material as a secondary hydration reaction [36]. The addition of biomass fly ash can have a retarding effect on the hydration reaction and, therefore, the quantitative analysis of the CH production and consumption during hydration and the amorphous structure are explored in our study.

Currently, it is worth noting that, unlike previous studies, in this study, not only was the microscopic characterization of biomass fly ash analyzed using SEM (EDS) but it was also co-analyzed with the results of particle size distribution testing. The effect of biomass fly ash particle properties, fineness, and homogeneity on the reactivity of pozzolanic material was investigated. The texture anisotropy of fly ash is influenced by different combustion conditions and raw material variations in biomass combustion ash. Its uneven form and heterogeneous structure may restrict fly ash’s ability to participate in a reaction as a pozzolanic specialty material [13]. Secondly, a comparative analysis of the composition of biomass fly ash and Class F fly ash was also carried out in this study to investigate the effect of their chemical composition on the participation of biomass fly ash in the hydration reaction. Our research investigated biomass fly ash collected without pretreatment, which was found to be uniform in shape and possess physical and chemical qualities equivalent to Class F fly ash, originating from a biomass power plant located in Gifu, central Japan. According to J. Jakub U. Malgorzata [13], the strength of concrete is not adversely impacted by the incorporation of 30% fly ash. However, there is a modest increase in water absorption at this percentage [24]. To be able to have a clearer view of the reactivity, mechanical properties, pore development, and deformation stability of biomass fly ash from a particular area of Japan, at different substitution rates, a more detailed division of the substitution rate has been made by setting it at 10%, 15%, 20%, 25%, and 30% to obtain a better choice between 10 and 30%. From a more environmentally friendly and economical perspective, if this region’s biomass fly ash can meet Japanese Industrial Standards without preprocessing, it would reduce costs further, enhance environmental impact, and increase the utilization rate of biomass energy.

It was found that the physicochemical properties of biomass fly ash closely resemble those of Class F fly ash and this was further explored. The primary objective of this study is to validate and substantiate the potential substitution of biomass fly ash for conventional cement fly ash supplementary cementitious material. By evaluating the pozzolanic reaction performance, compressive strength, mechanical characteristics, the drying shrinkage performance of concrete incorporating this biomass material, and the best replacement rate of this unmodified biomass fly ash for cement were estimated.

## 2. Materials and Experimental Program

### 2.1. Experimental Materials

The cement used was Ordinary Portland Cement (OPC) for the manufacture of hardened cement paste and mortar. And sea sand (S, density: 2.59 g/cm^3^, water absorpton: 0.76%, fineness modulus: 2.4, grain size: 0.08–0.16 mm) was used as fine aggregate and was conformed to JIS A 5308 [37]. A kind of woody biomass fly ash and Class F fly ash according to ASTM C618 [38] were used as SCMs in this experiment. Table 1 shows the physical characteristics of fly ash, biomass fly ash, and cement. Table 2 lists the chemical composition and Table 3 shows the mineral composition.

#### Fly Ash Characteristics

**Figure 1:**
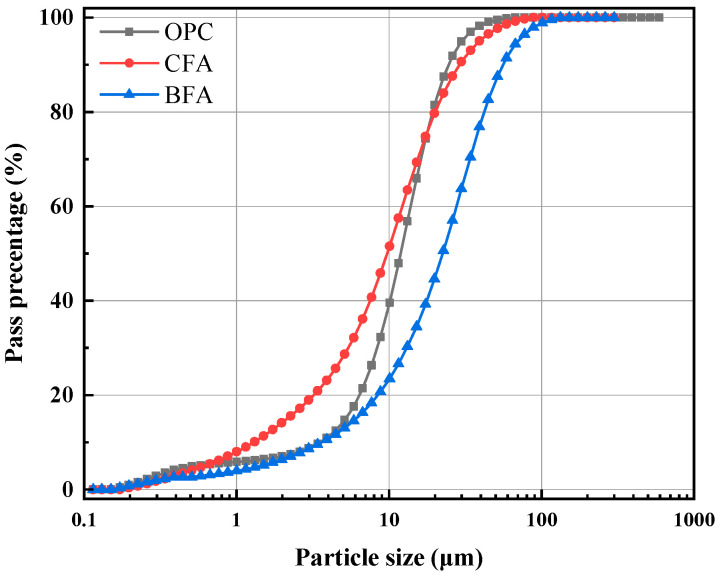
Particle size distribution of cement and fly ash.

Figure 1 demonstrates the particle size distribution test findings for the two kinds of fly ash and cement. Considering the experiment’s outcome, the CFA fly ash particles had a maximum particle size of 89 μm, while the total size of the particles of BFA fly ash was 133 μm. The peaks in BFA and CFA appeared between 10 and 50 μm, and the proportion of 10–100 μm in BFA was the highest, indicating that the predominant particle dimensions of two kinds of fly ash were 10–100 μm. And the median diameter (D50) was lowered from 17.74 μm (BFA) to 12.20 μm (CFA). Therefore, fly ash CFA displayed a somewhat more extraordinary fineness to fly ash BFA, but there were more significant numbers of particles of fly ash scattered in the interval of 10–50 μm. Compared with the results of previous experiments [39], it was observed that the particle dispersion characteristics of the biomass fly ash used in this study were remarkably similar to those examined by Katja Ohenoja et al. after the grinding process (fluidized bed opposed jet mill AFG operating at 18,000 rpm). The fineness performance of our biomass fly ash experiment was satisfactory, which may be due to the combustion mechanisms of biomass fuel in the power plant [23].

Table 1 displays the physical properties of the fly ashes. The fly ash CFA has a 2.21 g/cm^3^ density, while fly ash BFA was 2.41 g/cm^3^. However, the difference in densities between these two types of fly ashes is insignificant and did not exceed 9%. The pH values of fly ash CFA and BFA were 11.75 and 12.92, respectively. Loss on ignition (LOI) was measured by the weight loss of a material when it was heated to a high temperature, typically around 950 °C to 1000 °C. The obtained LOI values were shown. CFA showed an LOI value of 1.97% and a BFA of 1.59%. Table 1 reveals that the Blaine value of BFA is mildly smaller than that of CFA. The Blaine values of CFA and BFA were 4000 g/cm^2^ and 3540 g/cm^2^, representing an approximate difference of around 12%. This discrepancy could be attributed to variations in particle size between biomass fly ash (which falls within a range less than 10) and coal fly ash; consequently, the specific surface area of biomass fly ash tended to be somewhat lower due to dispersion effects on particle size [29]. The 91-day activity index indicated that specimens with BFA had satisfactory long-term pozzolanic reaction performance, consistent with Demis et al.‘s findings [40], suggesting good strength properties.

Table 2 displays the principal chemical analysis of the two types of fly ash using X-ray fluorescence. SiO_2_ was the major chemical component in the coal fly ash (CFA), followed by Al_2_O_3_, Fe_2_O_3_, CaO, and K₂O. SiO_2_, Fe_2_O_3_, and Al_2_O_3_ content accounted for more than 80%. Biomass fly ash contained typical wood-burning ash substances; SiO_2_ was the predominant chemical component in the biomass fly ash (BFA), followed by CaO, Al_2_O_3_, Fe_2_O_3_, K₂O, and MgO [22]. When biomass fly ash was used in place of Portland cement, the generated Portlandite reacted with water or silicates in the ash to produce products similar to those formed during cement hydration. The primary oxides (SiO_2_, Al_2_O_3_, and Fe_2_O_3_) in the biomass fly ash used in this experiment were higher than 70%; however, previous studies on woody biomass fly ash showed values below this [40]. The sum of the two active oxides, Al_2_O_3_ and Fe_2_O_3_ (22.6%), exceeded the empirical value (9%) in Ref [41]. Typically, small amounts of magnesium oxide promote the formation of the alite (C_3_S) [42]. The MgO content in the biomass fly ash was slightly higher than in CFA and below the 4–5% range, suggesting a slight effect on strength, consistent with the literature [41]. This variation could be attributed to the type of unprocessed biomass fuel and the combustion mechanism [23]. The high content of primary oxides in the biomass fly ash used in this study indicated its potential as an admixture with pozzolanic properties in concrete production, suggesting an optimal substitution rate to be explored.

Table 3 shows the X-ray diffraction data for fly ash’s primary crystallographic phases. Fly ash CFA contained 10.9% quartz and 14.6% mullite. In contrast, fly ash BFA had higher contents of quartz (23.3%), larnite (7.8%), and magnetite (6.7%). Silicon oxide mainly existed in an amorphous structure. Increased crystalline quartz and decreased SiO_2_ content in BFA typically reduced its pozzolanic reactivity [43]. Quartz, present as large, hard grains, often fills pores in the matrix [44]. The results indicated that BFA’s potential hydraulic properties were lower than CFA due to the lack of high calcium oxide phases (e.g., bassanite, gypsum, and free lime). Magnetite content was significantly higher in BFA than in CFA, consistent with literature findings [44]. Magnetite, an iron oxide mineral, was abundant in wood combustion ash due to iron oxide generation during combustion. This matched the iron oxide content results in Table 2. Periclase was another mineral present in both CFA and BFA, likely formed from dolomite decomposition. In woody combustion ash, it may be a residue of magnesium from organic matter. High MgO content in Table 2 supported this observation.

**Figure 2:**
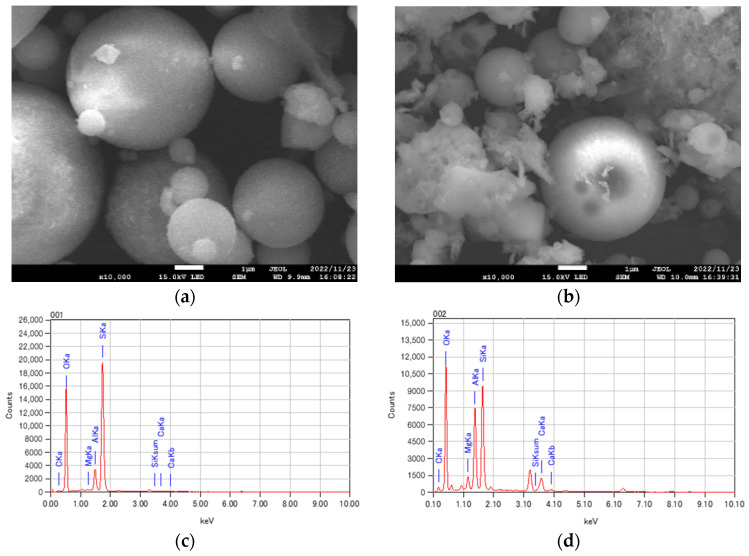
The SEM and EDS analysis of fly ash. (**a**) CFA fly ash SEM image; (**b**) BFA fly ash SEM image; (**c**) CFA fly ash EDS analysis; (**d**) BFA fly ash EDS analysis.

The SEM and EDS test results are indicated in Figure 2. The microstructure of CFA fly ash (Figure 2a) was relatively regular and spherical, with a smooth surface and no excess unburned carbon or impurities adhering to it (Figure 2c). In contrast, Figure 2b shows a micrograph of BFA fly ash displaying flaky irregularities, unlike previous studies [45]. However, the BFA used in this study mainly consisted of spherical particles, with only a small fraction being irregularly shaped. These irregular particles may increase roughness during blending, potentially reducing workability. Both types of fly ash had similar elemental compositions, primarily composed of Ca, Si, and Al, as shown in Figure 2c,d, supporting the findings from chemical composition tests (Table 2). Thus, the incorporation of biomass fly ash in this experiment showed promise as a viable substitute for cementitious materials.

### 2.2. Preparation of Cement Paste and Mortar

The cement paste and mortar mixing are described in Table 4 and Table 5. Ordinary Portland Cement (OPC), water and woody biomass fly ash, and sea sand were utilized. The cement paste and mortar mixing are shown in Table 4 and Table 5. In the experiments, 0.5 was selected for the water–binder ratio (w/b), and the replacement rates by mass of biomass fly ash BFA and Class F fly ash CFA were 10%, 15%, 20%, 25%, and 30%, respectively. All mixing proportions conform to JIS A6201:2015 [46]. The cement slurry is kneaded by adding a mixture of water, cement, and fly ash for 2.5 min. The pastes and mortars were cured, maintaining 20 ± 2 °C and 60 ± 10% relative moisture until the target material age (7, 28, and 91 days). Specimens were crushed at the target age. The samples obtained by acetone immersion to stop hydration were subjected to subsequent experiments.

### 2.3. Research Methods

The sample was added to the Laser Diffraction and Scattering Particle Size Analyzer and shaken for one minute and then the particle size distribution was automatically measured. The activity index test method covered by this study was based on JIS A 6021:2015 [46]. In this test method, the compressive strength of mortar mixed with Ordinary Portland Cement only (reference mortar) and mortar mixed with a binder of 75wt% of cement and 25wt% replaced by fly ash (test mortar) were substituted into Equation (1) to obtain the targeted material age activity index. The following equation is used to determine the activity index:

Activity index = C_1_/C_2_ × 100 (1)

where:

C_1_: Compressive strength of the test mortar at target age.
C_2_: Compressive strength of reference mortar at target age.

An X-ray fluorescence spectrometer (XRF) was applied to assess the chemical components of fly ash according to the standard “JIS K 0119:2008 General rules for X-ray fluorescence analysis” [47]. The heat loss of each ash corrected the quantitative values of chemical composition. Microstructures were imaged adopting SEM-EDS using a field emission scanning electron microscope (FE-SEM) and an energy dispersive spectrometer (EDS). For SEM-EDS measurements, the sample powder was placed in a specific mold into the instrument’s operating table and the microscopic properties of the sample were subsequently measured using an analytical instrument, the Zeiss Field Emission Scanning Electron Microscope (JEOL Ltd., Tokyo, Japan.). The mineralogical components were examined utilizing an XRD/Rietveld (X-ray diffraction) method with a fully automatic horizontal multipurpose X-ray diffractometer and the test was carried out at room temperature. The test light source was X-ray tube copper target radiation with a tube voltage of 40 kV, a current of 30 mA, and a scanning range of 5~63 deg. 2 θ. And this was analyzed using the analytical software pdxl2 (SmartLab Studio II, v4.0), which is used as an internal standard sample for analytical samples of α–10% mixture of Al_2_O_3_ (0.27 g) α. The calculated values of “unknown” were determined as “glass” [48]. The low heat loss of each ash corrected all the computed values. All of the above experiments, including the detailed methods of determining the physical properties of the materials, are based on these two standards: JIS A 6201 [46] and JIS R 5210 [49]. Rounding to one decimal place and correction of numerical errors were conducted according to JIS Z 8401 [50]. 

The CH content was determined using the thermogravimetric analyzer (TG). In this test, we used thermal analysis equipment to test the calcium hydroxide quantity, which effectively removes the influence of free water binding in the hydration process. Cement paste specimens were examined at 7, 28, and 91 days of age according to the following equations [51]. Calcium hydroxide content was obtained according to Equation (2):

CH content = WL × 74/18/Mc(2)

where:

The weight loss at 420–550 °C is denoted by WL;
The weight of the cement paste powder sample is Mc (mg);
The calcium hydroxide content is CH (wt%).

According to previous reports, the amorphous content was calculated from the measurement results of α-Al_2_O_3_ by Equation (3), as in Hoshino et al. [52]. Given that most of the amorphous components in hydrated cement paste are calcium silicate hydrate (C-S-H), this report considers this. The amorphous mass obtained from Equation (3) is defined as the total number of C-S-H.

GM = 100 · (G − GR)/{GA · (100 − GR)/100}(3)

where:

GM is the amorphous phase (C-S-H) (mass%).
GR is the α-Al_2_O_3_ mixture (mass%).
GA is the measured value of α-Al_2_O_3_ (mass%).

The mortar made for testing according to the mix proportions stated in Table 5 was measured for the strength of compression, drying shrinkage, and pore volume. To ascertain the compression strength of specimens, JIS A 1108’s “Compressive Strength Test Method of Concrete” was implemented [53]. The compressive strength test was performed with a cylinder (Ø = 100 mm × h = 200 mm). For each series, plastic molds were used for casting 12 cylinders and before demolding, stored in a chamber at 20 °C and 60% relative humidity for 24 h. JIS A 1129-2 “Method of Measuring Length Change of Mortar and Concrete” was applied to measure and calculate drying shrinkage [54]. A test specimen for dry shrinkage test using a cuboid of 40 × 40 × 160 mm was prepared. The cuboids were cast in a steel model for each combination and demolded after 24 h in a chamber at 20 ± 2.0 °C. The specimens were demolded after 24 h and cured in water at 20 ± 2.0 °C for 7 days before initial length measurements were taken. The initial length of the mortar sample was measured using a standard-length measuring instrument. The mortar specimen was placed horizontally on the measuring platform. The dial gauge was gently touched to one end of the specimen, ensuring that the probe was perpendicular to the surface of the specimen and made good contact. The dial gauge was recorded as the initial length. After the initial measurement was completed, the samples were cured at a temperature of 20 ± 2 °C and relative humidity of 60 ± 5%. At the target age, the length of the sample was measured until the length change stabilized. The mercury intrusion porosimetry (MIP) approach determined the pore structure and volume. For the sample of pore size distribution, a mortar cylinder of Ø 50 × 100 mm was prepared. After curing, the mortar specimens, from the central part of the mortar to limit data dispersion, were sliced into 5 mm thick fragments [46]. The prepared sample was used after it was immersed in acetone to stop the reaction and dried in a vacuum dryer. MIP was performed on an AutoPore V with a maximum sample carrying the pressure of 60,000 psi.

## 3. Experimental Results and Discussion

### 3.1. Thermal Analysis

**Figure 3:**
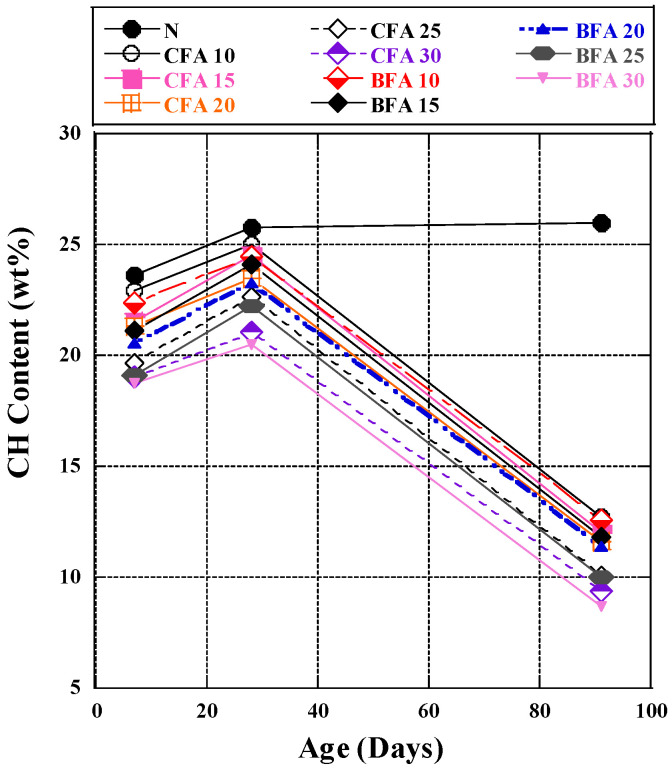
The Ca(OH)_2_ content of cement paste.

Based on the thermal analysis, the calcium hydroxide amount in the CFA and BFA mixtures is presented in Figure 3. During cement hydration, CH forms as a hydration product. The consumption of CH indicates the involvement of fly ash in the pozzolanic reaction [55]. The test results showed an increase in CH content from 7 to 28 days due to intense early hydration reactions [56]. Initially, OPC hydrates and delays the pozzolanic reaction by providing fly ash. From 28 to 91 days, CH levels decreased due to slower hydration reactions, as substantial quantities of CH generated were consumed by pozzolanic materials (FA), promoting more intense later-stage reactions [57].

For CFA fly ash mixtures, calcium hydroxide (CH) content decreased as the replacement rate increased from 7 to 91 days. In CFA-10, CH increased by 9% between 7 and 28 days, then decreased by 49% by 91 days. In CFA-20, CH increased by 10% from 7 to 28 days, then decreased by 50.5% by 91 days. In CFA-30, CH increased by 10.5% between 7 and 28 days, then decreased by 54.5% at 91 days. This trend is due to CH consumption in the pozzolanic reaction with fly ash, aligning with previous research [58]. Similar patterns were observed in BFA fly ash mixtures. In BFA-10, CH increased by 9.5% between 7 and 28 days, then decreased by 49.8% by 91 days. In BFA-20, CH increased by 13% from 7 to 28 days, then decreased by 56.5% by 91 days. In BFA-30, CH increased by 9.3% from 7 to 28 days, then decreased by 47.9% by 91 days. Initially, the effect of incorporating BFA was negligible but showed significant delayed progression later, especially at a 20% substitution rate. At seven days, CH content in BFA samples was lower than in CFA samples, suggesting later BFA incorporation into the reaction. By 28 days, BFA showed significant reactivity with CH. Compared to previous studies [59], BFA in this study displayed higher reactivity at a 20% substitution rate. From 28 to 91 days, CH decreased from 23.3% to 11.6%, while reference testing showed a decrease from 16.0% to 15.2%, highlighting BFA’s reactivity advantage. This hydration reaction produced CH for the pozzolanic reaction with fly ash to create C-S-H gel, contributing to compressive strength development. 

**Figure 4:**
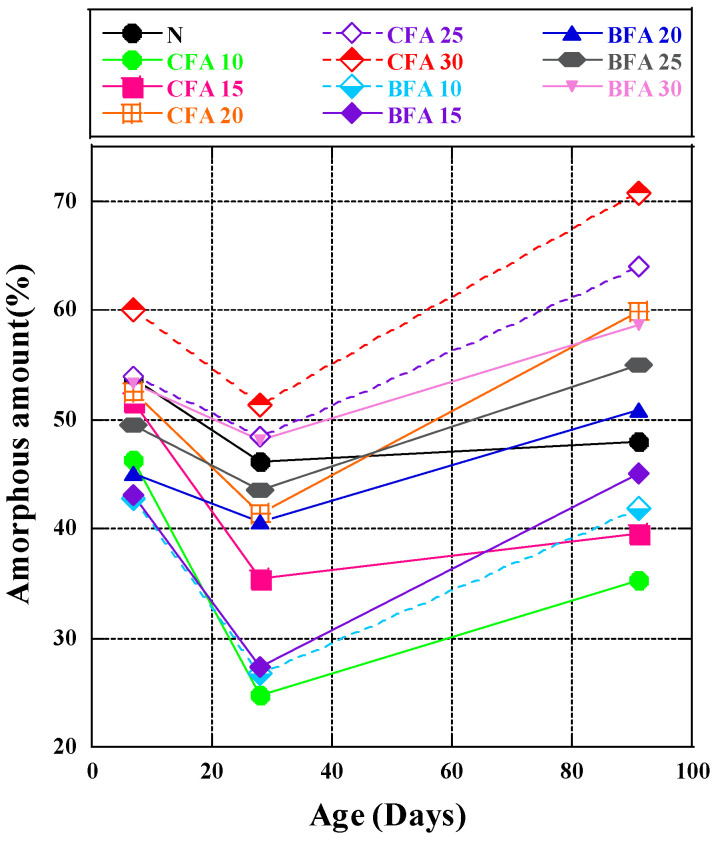
The amorphous amount of cement paste.

The linear regression formula demonstrated the relationship between calcium hydroxide (CH) content and compressive strength at 7, 28, and 91 days of curing (Figure 4). The coefficient of determination indicated CH content’s influence on compressive strength changes. After seven days, a strong correlation (R^2^ = 0.92) showed a significant impact of CH on compressive strength. At 28 days, the positive correlation remained strong (R^2^ = 0.94), indicating increased compressive strength with higher CH concentration. However, at 91 days, the correlation weakened (R^2^ = 0.63), suggesting CH content was less critical for compressive strength, with pore structure development becoming more influential [60]. Thus, CH content is crucial for assessing compressive strength in the early and middle hydration stages, with similar effects observed in cement slurry mixed with BFA.

In summary, the thermal analysis of CFA and BFA mixtures reveals emerging trends in cement hydration. The increase in calcium hydroxide (CH) content from 7 to 28 days indicates vigorous early hydration, followed by a decline from 28 to 91 days, signifying a slower but more intense pozzolanic reaction. Increasing fly ash admixture leads to reduced CH in CFA and BFA mixtures, emphasizing pozzolanic consumption. CH significantly influences compressive strength up to 28 days but, after 91 days, other factors, notably pore structure development, become more significant [61]. Interestingly, the impacts of BFA substitute cement in the paste are nearly identical to that of CFA in promoting pozzolanic reaction at the later stage, especially the replacement rate of 10–20%, which can achieve relatively favorable outcomes. The hydration reaction results confirmed the strong pozzolanic responsiveness of the biomass fly ash, which was utilized in this work.

### 3.2. Amorphous Phases Analysis

Figure 4 shows the production of amorphous phases, specifically calcium silicate hydrate (C-S-H) material, in cement paste. This material has a substantial Blaine value and positively impacts the strength and characteristics of the cement paste [60]. The test results (Figure 4) showed a significant reduction in amorphous content in all ratios using two different fly ashes from 7 to 28 days, followed by a rebound by 91 days, albeit to varying degrees. According to research [44], the amorphous phases measured included C-S-H gel and pozzolanic material in the reaction. This study quantitatively examined the amorphous phases based on C-S-H formed through hydration and unreacted fly ash. In the initial stages of hydration, cement clinker hydrated a significant quantity of C_3_S, generating a substantial volume of C-S-H gel within seven days. As the hydration reaction progressed into its intermediate stage, the other reactant of C-S-H, namely C_2_S, reacted slowly until 28 days and then began to sediment continuously across the surface of fly ash particles. A substantial quantity of fly ash involved in secondary hydration was consumed and the addition of fly ash delayed the hydration reaction to some extent [61]. We assumed that not only the reaction-generated amorphous phase is present in the whole system involved in the hydration reaction but also part of the amorphous phase is present in the fly ash involved in the reaction [62]. When the hydration reaction occurs, part of the amorphous phase in the fly ash is converted to the crystalline phase (CaO + H_2_O → Ca(OH)_2_), which increases significantly in this step [63]. However, the addition of fly ash does not sufficiently react as pozzolanic in the early stages, so the overall amorphous content decreases. Therefore, from 7 to 28 days, the quantity of amorphous material significantly declined. During the intermediate and late phases of the hydration reaction, the degree of C_2_S involved in the process gradually increased with age. The active substances (Fe_2_O_3_ and Al_2_O_3_) present in fly ash, as well as accumulated CH on its surface, underwent secondary hydration, leading to substantial quantities of C-S-H gel formation [64]. This result aligned with the Ca(OH)_2_ content test results. The combined data from the CH content test and the amorphous structure content test showed that biomass fly ash consumed a large amount of CH in the intermediate and final phases of the reaction (28 days) to generate a C-S-H amorphous structure. The capabilities were nearly identical to those of standard fly ash, highlighting the promoting effect of biomass fly ash on the gelation reaction with CH for C-S-H formation. In this experiment, according to the method of Seiichi Hoshino et al. [44], we first applied this method to the experiment of biomass fly ash participating in the pozzolanic reaction. By calculating the amorphous content and incorporating changes in CH amounts, quantitative analysis of C-S-H was achieved.

In CFA fly ash mixtures, the amorphous C-S-H generally showed an increasing trend with a rising fly ash replacement rate. For mixtures of fly ash and BFA, the C-S-H content varied significantly from 28 to 91 days. The major minerals, tricalcium silicate (C_3_S) and dicalcium silicate (C_2_S), in cement clinker are the primary sources of C-S-H gels during hydration [64]. This investigation employed two types of fly ash, which do not hydrate on their own under normal circumstances but consume CH emitted by clinker during the hydration reaction, leading to secondary hydration. However, previous findings [65] indicated that well-structured C-S-H was generated in 30% fly ash replacement samples within seven days and the C-S-H quantity did not change significantly after 30 days. This may be due to the excellent fineness of the fly ash BFA used in our experiment (particle size range 10–50 μm, with fly ash CFA at 50.7% and fly ash BFA at 61.9%). The fineness of fly ash contributes to the overall system’s particle fineness, helping the active ingredients in fly ash dissolve more readily and promoting the hydration of cement clinker [66]. 

The study observed a substantial reduction in amorphous content from 7 to 28 days across various fly ash ratios, rebounding by 91 days. Active substances in biomass fly ash contributed significantly to the formation of C-S-H gel, consuming a large amount of CH during the intermediate and late stages. CFA fly ash mixtures showed an increasing trend in amorphous C-S-H with increasing replacement rates, while BFA blends maintained consistent levels, particularly for 28–91 days. The fineness of BFA contributed to enhanced performance in promoting hydration reactions. Therefore, the chemical composition of the BFA tested in this experiment contains more CaO, MgO, more reactive components of pozzolan, and high-quality fineness, which can facilitate the synthesis of more C-S-H and potentially allow the growth of compressive strength.

### 3.3. Compressive Strength

**Figure 5:**
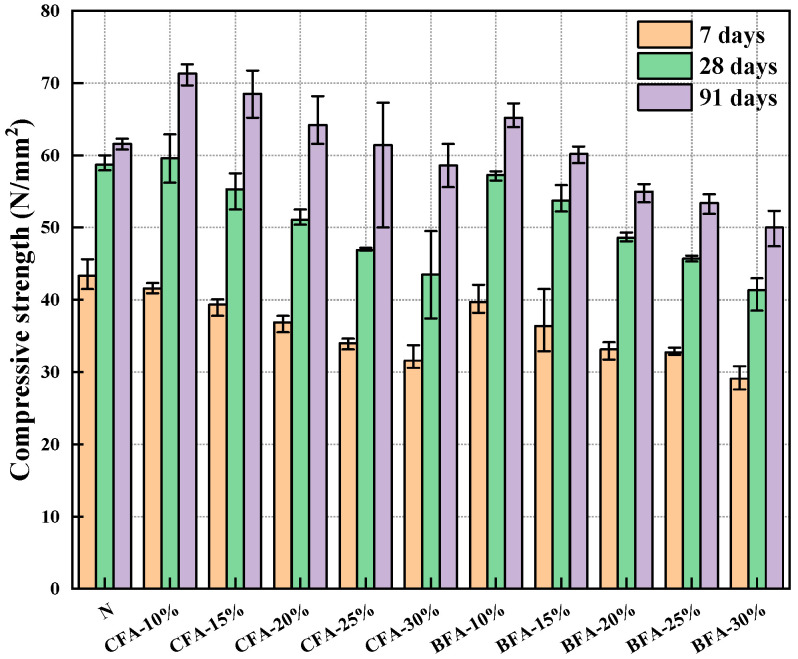
Compressive strength.

Figure 5 depicts the findings of the compressive strength of mortar specimens tested by adding CFA and BFA as cement substitutes into the mixture. The test results revealed that the compressive strength from the CFA-included mixture decreased between 7 and 28 days, with similar outcomes observed from 28 to 91 days. The findings demonstrated that the rate of compressive strength increase slowed down as the specimens aged during the first 28 days [67]. In CFA-10, the compressive strength increased by 41.6 MPa within the first seven days and by 18 MPa from day 7 to day 28. Similarly, in CFA-20, the increase in compressive strength was 36.9 MPa in the first seven days and 14.2 MPa from 7 to 28 days. In CFA-30, the compressive strength exhibited an increment of 31.6 MPa within the first seven days followed by a further increase of 11.9 MPa from day 7 to day 28. The results from the three groups of different CFA fly ash incorporation rates indicated that the growth of compressive strength gradually slowed with age in the first 28 days. This phenomenon might be a consequence of fly ash’s retarding effect on hydration and the slower appearance of hydration reaction products contributing to strength in the early stages [67]. As the quantity of CFA substitution increased, the compressive strength continued to decline. Between 7 and 28 days, none of the components matched the performance of the control group, except for CFA-10, which exhibited slightly higher strength. However, by 91 days, CFA replacement rates up to 25% surpassed the control group in compressive strength. This suggests that CFA performed well, possibly due to its favorable fineness and specific surface area properties, which positively influenced compressive strength development [68].

**Figure 6:**
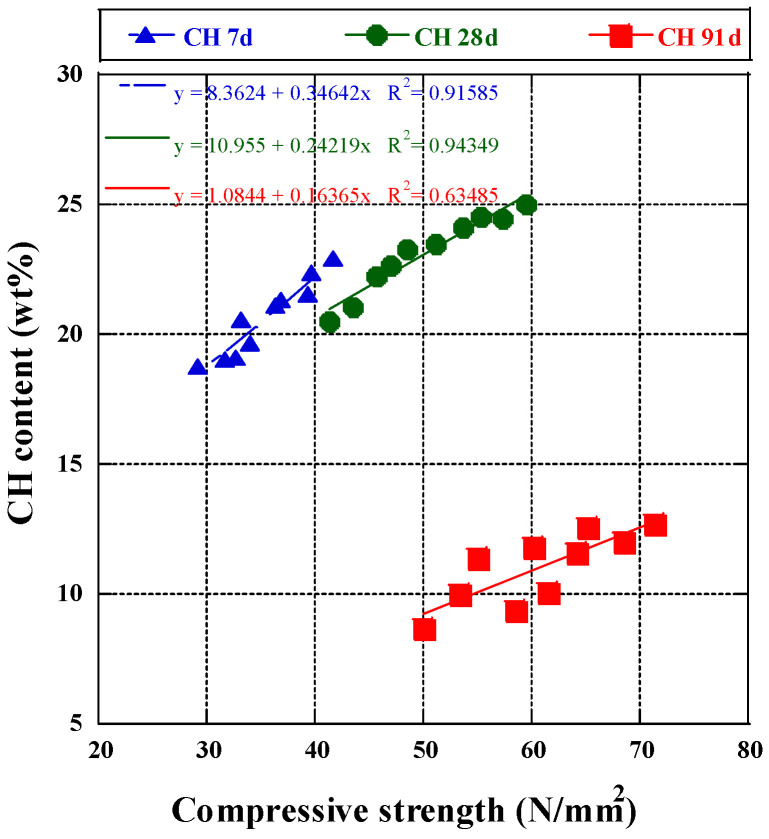
CH content and compressive strength regression curve.

Upon observation, the compressive strength tests with BFA fly ash incorporated into the mix yielded results similar to those with CFA fly ash. Compressive strength decreased with age between 7 and 28 days in the BFA-incorporated mixture, with similar outcomes observed from 28 to 91 days, showing gradual and slow growth. In BFA-10, the increase in compressive strength was 39.7 MPa in the first seven days and 17.6 MPa from 7 to 28 days. In BFA-20, the increase was 33.1 MPa in the first seven days and 15.5 MPa from 7 to 28 days. In BFA-30, the increase was 29.1 MPa in the first seven days and 12.2 MPa between 7 and 28 days. According to the results of V. Sata et al. [69], under experimental parameters with a W/B ratio of 0.5% and a biomass fly ash admixture of 20%, compressive strength rose from 6.2 MPa (7 days) to 10.2 MPa (90 days), significantly lower than the findings of this experiment (33.1 MPa at 7 days and 55.0 MPa at 91 days). This difference may be attributed to the superior fineness [70] and the pozzolanic reactive oxides [61] (alumina and iron oxides) in the BFA in this work were also much higher than the above references. Comparison of the 7- and 28-day compressive strengths for the same incorporation rates (10–30%) of modified biomass fly ash [71], showed significantly lower results than those of this experiment (e.g., 33.6 MPa at 7 days and 39.2 MPa at 28 days for a 10% substitution rate; 32.2 MPa at 7 days and 38.2 MPa at 28 days for a 20% substitution rate; and 22.6 MPa at 7 days and 26.7 MPa at 28 days for a 30% substitution rate). The emergence of good compressive strength may be attributed to the significant difference in active oxide content of biomass fly ash between this study and the literature (Al_2_O_3_: 6.51%; Fe_2_O_3_: 2.15%). The contribution of BFA to compressive strength growth at a later stage is insignificant. During the initial phase of hydration, a considerable quantity of Ca(OH)_2_ is produced, which has important implications for the development of early strength (7 and 28 days). The subsequent reaction of pozzolanic-active compounds in fly ash yields the C-S-H content that fills the capillary pores and facilitates the improvement of long-term strength [72]. Additionally, compressive strength dropped as more BFA fly ash was applied. The compressive strength of BFA-10 exhibited a 6% increase compared to the control group over 91 days. The compressive strength of BFA-15 was almost compared to that of the control group. BFA-10 is 5% lower than CFA-15 but 6.2% higher than CFA-25, both higher than the control group. The linear regression formula demonstrated the relationship between calcium hydroxide (CH) content and compressive strength at 7, 28, and 91 days of curing (Figure 6). The coefficient of determination indicated CH content’s influence on compressive strength changes. After seven days, a strong correlation (R^2^ = 0.92) showed a significant impact of CH on compressive strength. At 28 days, the positive correlation remained strong (R^2^ = 0.94), indicating increased compressive strength with higher CH concentration. However, at 91 days, the correlation weakened (R^2^ = 0.63), suggesting CH content was less critical for compressive strength, with pore structure development becoming more influential [73]. Thus, CH content is crucial for assessing compressive strength in the early and middle hydration stages, with similar effects observed in cement slurry mixed with BFA.

Compressive strength in CFA-included mixtures declined from 7 to 28 days, attributed to the retarding effect of fly ash on hydration. However, by 91 days, CFA replacement rates up to 25% outperformed the control group, showcasing its positive impact on compressive strength. BFA fly ash exhibited a similar pattern, with compressive strength decreasing between 7 and 28 days and gradually increasing thereafter. BFA-10 performed well, showing a 6% strength improvement over the control group by 91 days. Therefore, the BFA chosen for our experiment possessed excellent mechanical properties due to its well-pozzolanic properties. It achieved better compressive strength test results with a 10–15% BFA substitution rate compared with fly ash (CFA).

### 3.4. Drying Shrinkage

**Figure 7:**
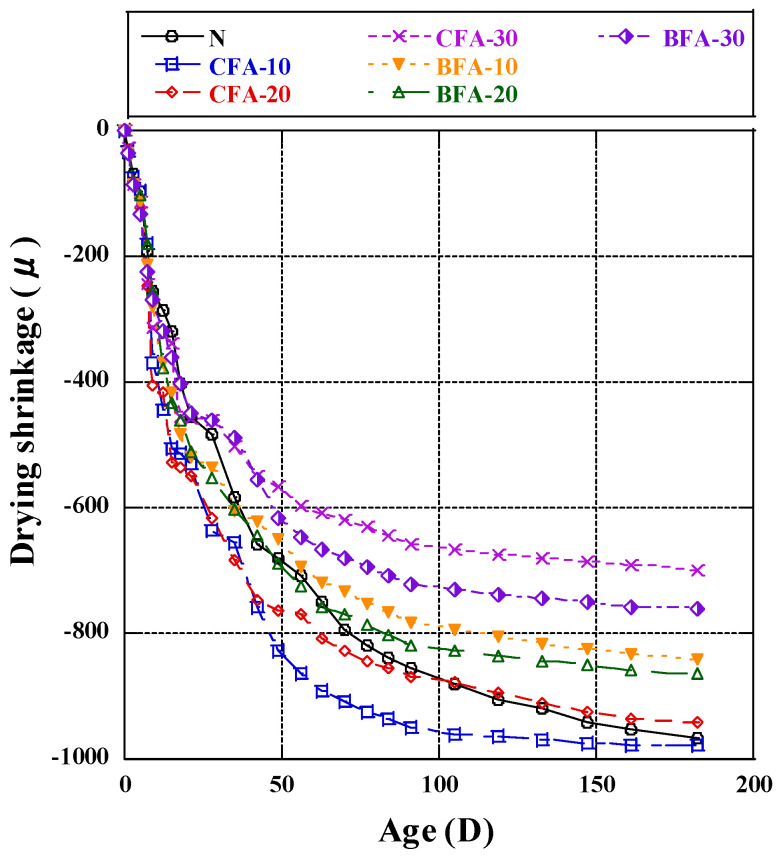
Drying shrinkage.

The test results of drying shrinkage of mortar specimens, made by adding CFA and BFA as cement substitutes into the blend, are shown in Figure 7. Drying shrinkage is considered the shrinkage strain resulting from the loss of physisorbed water in C-S-H gel, leading to crack formation and facilitating the ingress of aggressive agents into concrete, thereby compromising its long-term structural deformation stability [74]. As shown in Figure 7, in the mortar with CFA fly ash, drying shrinkage surged in the initial period (before 14 days), slowed down in the later period, and ceased to develop significantly after 56 days. As the ratio of fly ash replacement increased, drying shrinkage decreased considerably [75]. At a CFA substitution rate of 10%, the maximum drying shrinkage occurred. The drying shrinkage rate was 73.4% from 14 to 56 days in CFA-10, and the drying shrinkage growth rate decreased to 6.4% after 56 days. Among the specimens with CFA, those containing a 30% substitution rate exhibited the least drying shrinkage and were significantly more durable than the others. The drying shrinkage rate was 33.2% from 14 to 56 days in CFA-30, and the drying shrinkage growth rate decreased to 8.2% after 56 days. In mortar with BFA fly ash (Figure 7), drying shrinkage initially increased over the first 14 days but then increased slowly in the later period. The sample with a 30% substitution rate of BFA fly ash exhibited the least drying shrinkage and the best deformation stability, although the difference was insignificant. The maximum drying shrinkage occurred at the BFA fly ash replacement rate of 20%. The drying shrinkage rate was 65.7% from 14 to 56 days in BFA-30, and the drying shrinkage growth rate decreased to 8.4% after 56 days. In the initial phase, the incorporation of BFA fly ash outperformed CFA fly ash regarding drying shrinkage inhibition.

In mortars with varying ratios of two types of fly ash, drying shrinkage increased rapidly in the first 14 days but slowed down later in all mixtures. The minimum drying shrinkage was observed at a 30% CFA fly ash replacement rate, while the maximum change occurred at a 10% rate of CFA fly ash substitution. This could be due to the lower reaction (both of hydration and pozzolanic reaction) of blended cement and the dilution effect. Generally, drying shrinkage was suppressed as the replacement rate of fly ash increased [76]. Additionally, we found that the drying shrinkage was slightly more significant than that of the best-performing samples when using a 30% BFA fly ash substitution rate. However, samples with BFA fly ash all exhibited better performance in terms of drying shrinkage compared to those with CFA at 20% and 10% substitution rates. The drying shrinkage results of biomass fly ash incorporation in this study performed better [77], possibly due to the participation of highly reactive biomass fly ash in the pozzolanic reaction, leading to the generation of C-S-H gel and refining the development of pore size, which significantly influences shrinkage. Water loss and deformation are the primary causes of cement mortar shrinkage. Based on previous studies, this phenomenon may be related to the porosity of the samples as well as the water loss rate [78]. This conclusion is further supported by the test results of amorphous C-S-H content in this experiment. The amorphous structure content of BFA-30 is higher than that of CFA-10, refining the pore structure and inhibiting shrinkage caused by water loss deformation.

In this study, CFA-30 and BFA-30 exhibited the least drying shrinkage, enhancing long-term deformation stability. Higher fly ash replacement rates generally reduced drying shrinkage. Notably, BFA fly ash, especially at a 30% substitution rate, outperformed CFA fly ash in inhibiting drying shrinkage, attributed to its participation in pozzolanic reactions. The influence of fly ash dosage and the pozzolanic properties of the biomass fly ash used were also significant and essential concerning the drying shrinkage and deformation of mortar, while keeping the key factor, water–cement ratio, constant. The use of biomass fly ash in this study effectively promoted the generation of an amorphous gel structure, thereby improving the pore structure to inhibit drying shrinkage. Therefore, the difference in drying shrinkage in the study not only came from the difference in the substitution rate of fly ash but was also affected by the pozzolanic properties. The biomass fly ash used in our experiments performed well in terms of deformation stability.

### 3.5. Micro-Pore Structure

**Figure 8:**
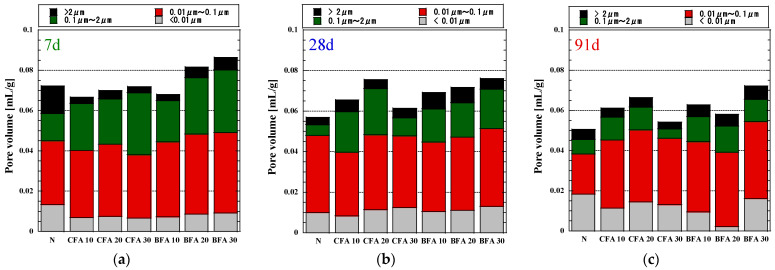
Cumulative pore volumes of the mortars. (**a**) Cumulative pore volumes of the mortars at 7 days; (**b**) Cumulative pore volumes of the mortars at 28 days; (**c**) Cumulative pore volumes of the mortars at 91 days.

The cumulative pore volume of the specimens into which fly ash was added is revealed in Figure 8. Following the test results, the pore volume growth of all illustrations diminishes with age. The inclusion of BFA slows down the cement hydration process compared to CFA, delaying the development of micropore structures in cement mortar. Although gel pores (less than 0.01 µm) in CFA and BFA are initially comparable, macropores (larger than 1 µm) increase, especially those exceeding 2 µm. The cumulative pore size for a 10% BFA replacement rate is like that of CFA at the same rate. The pore volume distributions depicted in Figure 8 illustrate the variation in pore size distribution with age for different fly ash replacement rates. The particle sizes and pozzolanic capabilities of fly ash significantly influence the porosity and pore dimensions of the mixes, depending on the source of the fly ashes [79]. As the material ages, the pore volume below 0.1 µm continues to increase, indicating the formation of more small pore structures despite the decrease in total pore volume, consistent with previous mortar studies [80]. The aging process appears to enhance the water tightness of biomass fly ash, resulting in a denser texture that enhances mortar’s deformation stability by preventing the accumulation of free water.

**Figure 9:**
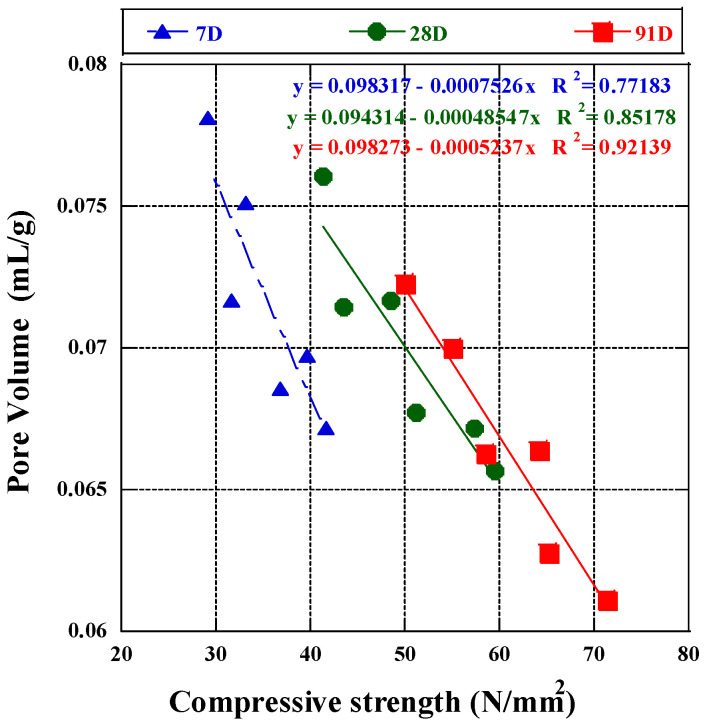
Pore volume and compressive strength regression line of fly ash at different ages.

**Figure 10:**
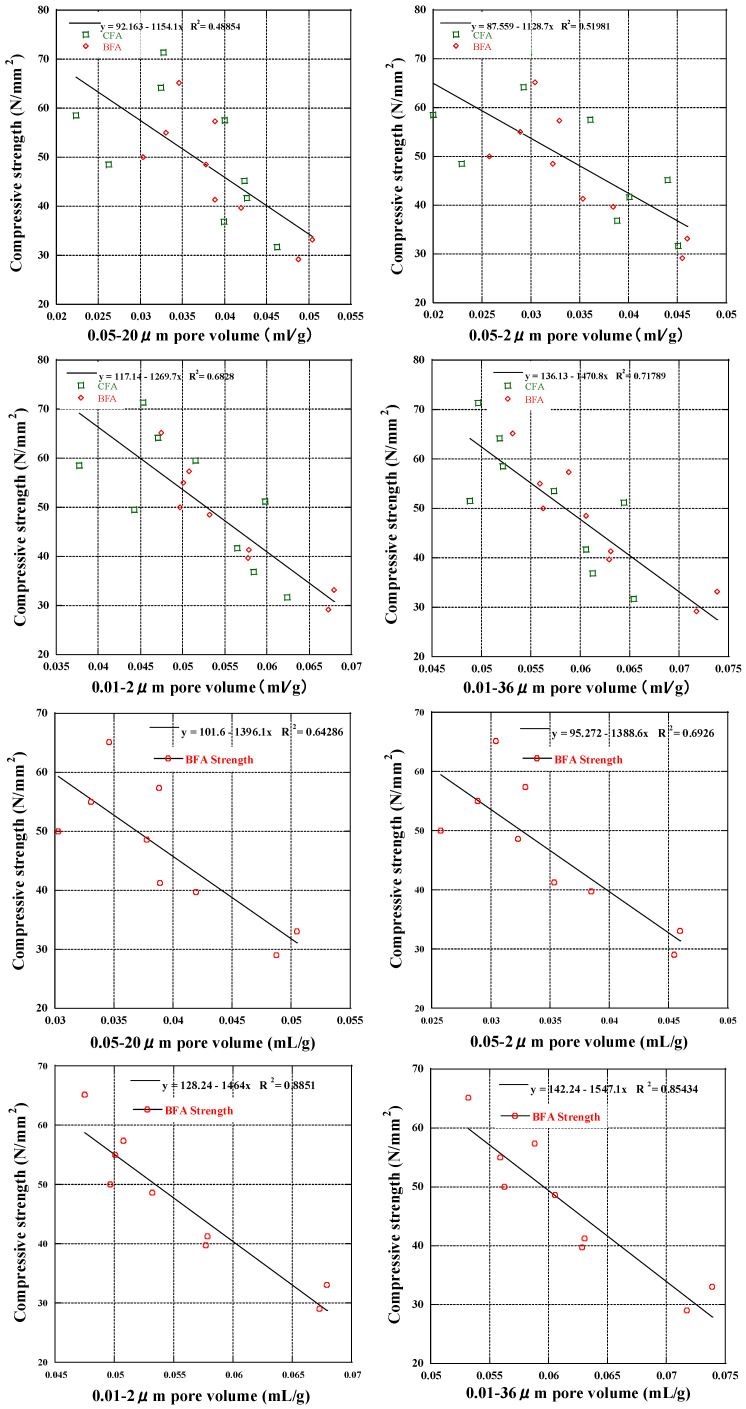
The correlation between pore volume and compressive strength.

Based on past findings, Xiao Han [81] and Wu and Lian [82] investigated that capillary pores (above 0.01 µm) had the greatest impact on strength, whereas gel pores (under 0.01 µm) had the most significant impact on deformation. We examined the correlation between pore volume and compressive strength across all ages and mix proportions using pore diameter and volume data. Three linear regression formulas were derived to depict this relationship in BFA mortar after 7, 28, and 91 days of curing, as shown in Figure 9. The fitting results revealed that porosity significantly influenced strength development, with varying degrees of influence observed at different ages. Specifically, after seven days of curing, the coefficient of correlation (R^2^ = 0.77) indicated a weak association between compressive strength and pore volume. This association intensified after 28 days (R^2^ = 0.85) and further strengthened after 91 days (R^2^ = 0.92), suggesting a positive regression. It was discovered that compressive strength increases as the pore structure refines and the mortar achieves greater density. There was high correlation with later compressive strength at 28 and 91 days, with linear correlation coefficients greater than 0.85 [60], indicating that pore volume at the latest age of hydration reaction was a key factor affecting strength. This aligns with our findings on the correlation between CH content and compressive strength, suggesting that the pozzolanic reaction in which BFA participates positively impacts strength development. To further clarify the effect of different pores on strength, we divided them according to different pore sizes and analyzed the correlation between pore volume and compressive strength. The coefficient of determination indicates that pore volume influences the change in compressive strength (Figure 10).

After further individual analysis of the BFA samples in the target pore size interval, BFA showed strong correlation. Consequently, the compressive strength was calculated for pore volumes greater than 0.01 or 0.05 µm (fine capillary pores). Pores larger than 0.05 µm had negligible influence on compressive strength, so this portion of the pores was not a significant factor in the strength, which was consistent with previous research [82]. Pores larger than 0.01 µm were linearly correlated with compressive strength, which suggests that pores bigger than 0.01 µm play a role in the progression of compressive strength. The effect of pores in the 0.01–36 µm section on strength was relatively weak, and further analysis showed that the maximum value of R^2^ between 0.01 and 2 µm is about 0.89. We conjecture that the pores in the 0.01–2 µm range are the primary elements promoting strength development, and we can also consider that the pores in this section are the pores that are harmful to strength. Teixeira et al. [28] observed the most pronounced differences in the 0.01–0.1 µm and 0.1–1 µm ranges, where, as the dose of BFA rose, the pore volume climbed. We believe that the shape of the fly ash particles contributed to the development of pores mostly and that capillary particles with highly irregular shapes might motivate the number of tiny pores.

As the results show, the addition of biomass fly ash (BFA) to cement mortar was found to delay the cement hydration process, resulting in slower development of micropore structures due to the homogeneity and excellent pozzolanic properties. Pore size distributions revealed an increase in small pores over time, suggesting improved water tightness and denser texture as the material ages. Linear regression analysis demonstrated a positive correlation between porosity and compressive strength, with a significant impact observed after 28 and 91 days of curing. Pores larger than 0.01 µm were identified as key contributors to strength development, while irregularly shaped fly ash particles were associated with increased pore volume. Overall, BFA showed the potential to enhance concrete deformation stability.

## 4. Conclusions

In our work, biomass fly ash from central Japan was analyzed and compared with Class F fly ash; fly ash samples were comprehensively described in terms of fundamental characteristics and particle morphology, chemical composition, and mineralogical properties. Emphasis was placed on the proper availability of biomass fly ash.

(1) The properties of BFA showed good performance, with activity test results comparable to Class F. The particles are regular, homogeneous, rich in active oxides, and have high pozzolanic activity. BFA demonstrated promising characteristics for concrete production, including good fineness, workability, and pozzolanic activity, which made it a potential supplementary cementitious material.
(2) Hydration reaction results confirmed the strong pozzolanic reactivity of the biomass fly ash used in our experiment. This reaction produced Ca(OH)_2_, which reacted with fly ash to form C-S-H gel, enhancing compressive strength. BFA substitution for cement at 10–20% showed similar or better results compared to CFA fly ash.
(3) With a 10–15% substitution rate of BFA, the compressive strength matched that of CFA in the initial phase (7–28 days), demonstrating similarly excellent mechanical properties. Although both CFA and BFA mixtures initially experienced a reduction in strength, they showed substantial improvement by 91 days, underscoring BFA’s beneficial influence on compressive strength. The pozzolanic activity of BFA also offered potential for further strength development.
(4) Drying shrinkage and pore development results confirmed that a 30% BFA substitution outperformed CFA in inhibiting shrinkage, indicating superior deformation stability. BFA-30 cement shows superior performance with a lower drying shrinkage rate of 65.7% from 14 to 56 days compared to CFA-10’s 73.4%. BFA delayed cement hydration, resulting in denser microstructures and improved water tightness, showcasing its potential in enhancing concrete stability.
(5) This study concluded that biomass fly ash, used without preprocessing, performed best at a 10–15% replacement rate. It enhanced mortar properties, with CaO boosting compressive strength through hydration and Al_2_O_3_ and Fe_2_O_3_ promoting pozzolanic reactions and C-S-H synthesis. BFA improved mechanical and deformation properties by generating C-S-H fillings and refining pores. BFA has similar prospects to CFA in its application as a supplementary cementitious material to replace OPC cement.

This study compared biomass fly ash (BFA) from central Japan with Class F fly ash (CFA). BFA demonstrated strong potential as a supplementary cementitious material due to its fineness, workability, and high pozzolanic activity. With a 10–15% substitution rate, BFA enhanced compressive strength and a 30% rate reduced drying shrinkage and improved deformation stability, making BFA a viable alternative to OPC cement.

## Figures and Tables

**Table 1:** Physical characteristics of fly ash, biomass fly ash, and cement.

Type	Density (g/cm^3^)	pH	LoI (%)	Blaine Value (g/cm^2^)	91 Day Activity Index (%)
OPC	3.16	-	0.80	3000	-
CFA	2.21	12	1.97	4000	90
BFA	2.41	13	1.59	3540	80

**Table 2:** The chemical component of fly ash, biomass fly ash, and cement.

Chemical Component (wt%)	OPC	CFA	BFA
SiO_2_	21.5	60.3	49.5
CaO	62.9	6.8	13.8
Al_2_O_3_	5.4	16.5	12.1
Fe_2_O_3_	3.0	8.0	10.5
K_2_O	1.1	1.7	5.7
MgO	2.1	1.6	2.9
TiO_2_	0.3	2.4	1.3
SO_3_	1.4	0.2	0.8
Others	1.5	0.8	1.4
LOI	0.8	1.6	2.0

**Table 3:** Mineral component of fly ashes.

Mineral Component (wt%)	CFA	BFA
Quartz low	10.9	23.3
Mullite	14.6	0
Periclase	0.9	2.2
Lime	0.2	0
Magnetite	0.3	6.7
Bassanite	0.9	0
Calcium cyclo-hex aluminate	0	0.2
Calcite	0.2	0
gypsum	1	0
Larnite	1.0	7.8
Ettringite	5.8	3.3
Calcium hydroxide	0.4	1.1
Brownmillerite	0.4	0.2
Hatrurite	0.1	0
Amorphous	63.3	55.1

**Table 4:** Mix proportion for cement paste.

Specimen Code	Unit Mass (kg/m^3^)			
	W/B	C	FA	W
N	0.5	1200	0	600
CFA 10		1080	120	600
CFA 15		1020	180	600
CFA 20		960	240	600
CFA 25		900	300	600
CFA 30		840	360	600
BFA 10		1080	120	600
BFA 15		1020	180	600
BFA 20		960	240	600
BFA 25		900	300	600
BFA 30		840	360	600

**Table 5:** Mix proportion for mortar.

Specimen Code	Unit Mass (kg/m^3^)				
	W/B	C	FA	W	S
N	0.5	500	0	250	1515
CFA 10		450	50	250	1508
CFA 15		425	75	250	1504
CFA 20		400	100	250	1501
CFA 25		375	125	250	1498
CFA 30		350	150	250	1494
BFA 10		450	50	250	1510
BFA 15		425	75	250	1507
BFA 20		400	100	250	1505
BFA 25		375	125	250	1502
BFA 30		350	150	250	1500

## Data Availability

The data presented in this study are available on request from the corresponding author. The data are not publicly available due to privacy restrictions.
